# Comparing haploidentical transplantation with post-transplantation cyclophosphamide and umbilical cord blood transplantation using targeted busulfan in children and adolescents with hematologic malignancies

**DOI:** 10.1007/s44313-025-00057-7

**Published:** 2025-01-23

**Authors:** Kyung Taek Hong, Bo Kyung Kim, Hong Yul An, Jung Yoon Choi, Sang Hoon Song, Kyung-Sang Yu, In-Jin Jang, Hyoung Jin Kang

**Affiliations:** 1 Department of Pediatrics, Seoul National University College of Medicine, Seoul National University Cancer Research Institute, Seoul, Republic of Korea; 2 Department of Laboratory Medicine, Seoul National University College of Medicine, Seoul, Republic of Korea; 3 Department of Clinical Pharmacology and Therapeutics, Seoul National University College of Medicine, Seoul, Republic of Korea; 4 Wide River Institute of Immunology, Seoul National University, Hongcheon, Republic of Korea

**Keywords:** Haploidentical, Post-transplant cyclophosphamide, Cord blood, Children, Hematologic malignancy

## Abstract

**Purpose:**

This study compared the outcomes of haploidentical-related donor (HRD) and umbilical cord blood (UCB) hematopoietic stem cell transplantation (HSCT) in pediatric patients with hematologic malignancies.

**Methods:**

Data on patients who underwent HRD HSCT with post-transplant cyclophosphamide (*n* = 41) and UCB HSCT (*n* = 24) after targeted busulfan-based myeloablative conditioning with intensive pharmacokinetic monitoring between 2009 and 2018 were retrospectively analyzed.

**Results:**

The median follow-up durations in the HRD and UCB groups were 7.0 and 10.9 years, respectively. The cumulative incidence of acute graft-versus-host disease (GVHD) grades II–IV and moderate-to-severe chronic GVHD did not differ significantly between the groups. However, the HRD group demonstrated significantly lower rates of acute GVHD grades III–IV (4.9% vs. 29.2%, *p* = 0.009) and non-relapse mortality (2.6% vs. 34.2%, *p* < 0.001) but a higher relapse incidence (32.1% vs. 8.8%, *p* = 0.004) than the UCB group. The 5-year event-free and overall survival rates were 65.8% and 54.2% (*p* = 0.204) and 78.0% and 65.7% (*p* = 0.142) for the HRD and UCB groups, respectively. Multivariate analysis identified disease status as a significant risk factor for overall survival (hazard ratio, 3.24; *p* = 0.016). Additionally, UCB HSCT exhibited a trend toward worse event-free survival compared to HRD HSCT (hazard ratio, 2.63; *p* = 0.05).

**Conclusions:**

These findings indicate that HRD HSCT with post-transplant cyclophosphamide provides promising outcomes compared to UCB HSCT in pediatric patients, with a trend toward improved survival over a long-term follow-up period exceeding a median of 7 years. Thus, HRD HSCT may be a valuable option for pediatric patients without human leukocyte antigen-matched donors.

**Supplementary Information:**

The online version contains supplementary material available at 10.1007/s44313-025-00057-7.

## Introduction

Haploidentical-related donor (HRD) and umbilical cord blood (UCB) stem cell sources are alternative donor strategies for hematopoietic stem cell transplantation (HSCT) when a matched sibling or unrelated donor is unavailable These alternatives are particularly valuable given that the likelihood of finding a matched unrelated donor varies significantly with ethnicity, ranging from 25 to 80% [1]. Recently, the use of UCB has declined in cases where a matched donor is unavailable, while HRD has become more widely adopted [2]. The advantages of UCB include a lower risk of chronic graft-versus-host disease (GVHD) and rapid graft availability. However, slower immune recovery and higher rates of opportunistic infections remain significant challenges [3–7]. In contrast, HRD HSCT approaches using either T-cell-depleted or T-cell-replete methods have shown promising outcomes, including high engraftment rates and low GVHD and non-relapse mortality (NRM) rates [8–11]. Among the T-cell-replete HRD HSCT methods, recent findings indicate that post-transplantation cyclophosphamide (PTCy) for GVHD prophylaxis improves leukemia- and GVHD-free survival compared with antithymocyte globulin (ATG) [12].

The choice between UCB and HRD in patients without suitable human leukocyte antigen (HLA)-matched donors remains unclear. While several studies have shown no significant differences in outcomes between UCB and HRD HSCT in adults with hematologic malignancies[13, 14], recent studies, including a prospective randomized trial, have shown better results for HRD in adults [15–18]. However, comparative studies in pediatric patients are limited. A retrospective study comparing T-cell-depleted, reduced-toxicity HRD HSCT with UCB HSCT in children with acute leukemia reported higher leukemia-free survival with HRD HSCT [19]. Similarly, Mo et al. observed improved clinical outcomes with HRD HSCT using ATG compared with UCB HSCT [20, 21]. In contrast, a Spanish multicenter study of pediatric acute myeloid leukemia reported comparable outcomes between HRD and UCB HSCT [22]. However, variations in treatment protocols and institutional differences complicate the ability to draw definitive conclusions.

Since 2009, our center has implemented a targeted busulfan-based myeloablative conditioning regimen with intensive pharmacokinetic (PK) monitoring and ATG-based GVHD prophylaxis for matched donors and UCB HSCT, which has demonstrated promising outcomes.[23] Additionally, we previously reported favorable results for HRD HSCT using PTCy-based GVHD prophylaxis in conjunction with this targeted busulfan-based conditioning approach [24]. Despite these advances, few studies have compared HRD HSCT with PTCy or UCB HSCT with ATG in pediatric and adolescent patients with hematological malignancies in Korea. Given the differing timelines for HRD and UCB HSCT at our institution, periodic comparisons of unrelated HSCT outcomes were performed to evaluate treatment differences. This study further investigated and compared the impact of graft source on outcomes in children and adolescents with hematologic malignancies following targeted busulfan-based myeloablative conditioning with PK monitoring.

## Methods

### Patients

The outcomes of 65 patients with high-risk hematologic malignancies who received their first allogeneic HSCT from HRD (n = 41) or UCB (double-unit, n = 21; single-unit, n = 3) at Seoul National University Children's Hospital between January 2009 and December 2018 were retrospectively analyzed. All patients underwent an intensive PK monitoring targeted busulfan-based myeloablative conditioning regimen.

UCB HSCT was conducted at our institution until 2016, whereas HRD HSCT with PTCy was initiated in 2014; therefore, the comparisons considered potential outcome differences due to timing. Consequently, the unrelated HSCT group (n = 87) served as the control group and was divided into two cohorts: the unrelated 09–13 (n = 51) and unrelated 14–18 (n = 36) groups, which received HSCT from 2009–2013 and 2014–2018, respectively. These groups were first compared as controls for temporal differences to facilitate comparisons between the HRD and UCB groups. During the overlap period from 2014 to 2016, UCB was prioritized when no suitable related or unrelated donors were available, with HRD selected if an appropriate UCB donor was not identified.

The Institutional Review Board of Seoul National University Hospital approved the procedure for reviewing the medical records and waived the requirement for obtaining consent (H-1107–024–368).

### Donor selection

When selecting unrelated donors or HRDs, HLA-A, HLA-B, HLA-C, and HLA-DRB1 matching was confirmed in all patients and donors using high-resolution molecular methods, with a preference for HRDs with the killer cell immunoglobulin-like receptor B haplotype. For UCB donor selection, HLA-A and -B serological typing and HLA-DR allele typing were performed.

### Transplantation protocol

All patients received busulfan-based myeloablative conditioning regimens. An initial dose of busulfan (120 mg/m^2^ for patients aged ≥ 1 year and 80 mg/m^2^ for those aged < 1 year) was administered intravenously on day −8. Subsequent doses were adjusted daily from days −7 to −5, based on the therapeutic drug monitoring results from the previous day. The total target area under the curve (AUC) for busulfan was set at 74–76 mg × h/L [23].

The HRD group received a conditioning regimen consisting of targeted busulfan, fludarabine (40 mg/m^2^ intravenously once daily from days −8 to −4), and cyclophosphamide (14.5 mg/kg intravenously once daily from days −3 to −2). Most patients in the UCB and unrelated groups received a regimen of targeted busulfan, fludarabine (40 mg/m^2^ intravenously once daily from days −8 to −4) ± etoposide (20 mg/kg intravenously once daily from days −4 to −2), along with ATG (thymoglobulin, Genzyme Transplant, Cambridge, MA; 2.5 mg/kg intravenously once daily from days −8 to −6 for the UCB group or −4 to −2 for the unrelated group).

For GVHD prophylaxis, the HRD group received PTCy (50 mg/kg intravenously once daily from days 3 to 4), along with tacrolimus and mycophenolate mofetil. The UCB group was treated with cyclosporine and mycophenolate mofetil, whereas the unrelated group received tacrolimus and methotrexate. Prophylactic treatments for veno-occlusive diseases and infections were administered according to institutional HSCT guidelines.[23]

### Definitions

Neutrophil engraftment was defined as the first of three consecutive days with a neutrophil count > 0.5 × 10⁹/L, while platelet engraftment was defined as the first of three consecutive days with a platelet count > 20 × 10⁹/L, without any transfusions for at least 7 days. Acute and chronic GVHD were diagnosed and graded according to standard criteria. [25, 26] Regimen-related toxicity, excluding GVHD, was graded up to 42 days post-transplantation using the National Cancer Institute Common Toxicity Criteria (v4.03).

### Statistical analyses

Categorical variables were compared using the chi-square test, while continuous variables were analyzed using Student's t-test or one-way analysis of variance. The incidence rates of relapse, NRM, and GVHD were calculated using the cumulative incidence function. In this context, the competing risk for relapse was NRM; the competing risk for NRM was relapse; and the competing risks for GVHD included graft failure and NRM. Events were defined as death, relapse, or graft failure. Survival analyses were performed using the Kaplan–Meier method. Differences in the cumulative incidence curves were assessed using Gray’s test, while differences in survival rates were evaluated using the log-rank test. A Cox proportional hazards regression model was employed for the multivariate analysis of prognostic factors affecting survival, utilizing the backward elimination method (*p* < 0.05), and included independent variables with *p *< 0.2. Statistical significance was set at *p* < 0.05. Statistical analyses were performed using R version 3.2.2 (www.r-project.org) and IBM SPSS Statistics for Windows, version 29.0.2.0 (IBM Corp., Armonk, NY, USA).

## Results

### Patient characteristics

The clinical characteristics of the patients in the UCB (*n *= 24) and HRD (*n* = 41) groups are summarized in Table 1. The median age at HSCT was significantly lower in the UCB group than in the HRD group (2.4 vs. 11.3 years old, *p* = 0.004). The distribution of diagnoses and remission statuses at HSCT did not differ significantly between the groups. All patients underwent targeted busulfan- and fludarabine-based myeloablative conditioning. Etoposide was administered to patients in the UCB group with acute lymphoblastic or high-risk acute myeloid leukemia. All patients in the HRD group received a regimen consisting of busulfan, fludarabine, and cyclophosphamide with peripheral blood as the stem cell source. All patients in the UCB group underwent double-unit UCB HSCT, except for three who received a single unit. The median follow-up durations did not differ significantly between the UCB and HRD groups (10.9 years vs. 7.0 years). Detailed patient characteristics and comparisons, including those of the unrelated groups, are presented in Supplementary Table 1.

**Table 1:** Patient characteristics

	UCB (n = 24)	HRD (n = 41)	*p*-value
Median age, years (IQR)	2.4 (1.4–6.1)	11.3 (5.8–14.2)	0.004
Sex, No. (%)			0.05
Male	8 (33.3%)	24 (58.5%)	
Female	16 (66.7%)	17 (41.5%)	
Median BSA, m^2^ (IQR)	0.55 (0.48–0.80)	1.11 (0.67–1.54)	< 0.001
Median body weight, kg (IQR)	12.23 (9.8–21.1)	31.2 (15.7–50.6)	< 0.001
Diagnosis, No. (%)			0.271
Acute lymphoblastic leukemia	10 (41.7%)	16 (39.0%)	
Acute myeloid leukemia	10 (41.7%)	14 (34.1%)	
Myelodysplastic syndrome^a^	0 (0.0%)	3 (7.3%)	
Malignant lymphoma	0 (0.0%)	6 (14.6%)	
Others^b^	4 (12.5%)	2 (4.9%)	
Conditioning regimen			< 0.001
Bu + Flu	8 (33.3%)	0 (0.0%)	
Bu + Flu + VP	14 (58.3%)	0 (0.0%)	
Bu + Mel + (Flu or Cy)	2 (8.3%)	0 (0.0%)	
Bu + Flu + Cy	0 (0.0%)	41 (100.0%)	
Status			0.993
CR1	17 (70.8%)	29 (70.7%)	
≥ CR2 or persistence	7 (29.2%)	12 (29.3%)	
Infused busulfan AUC, mg x h/L (IQR)	73.7 (72.0–75.2)	74.5 (74.0–76.0)	0.074
Median follow-up years (IQR)	10.9 (0.4–14.1)	7.0 (4.4–8.7)	0.318

*UCB* umbilical cord blood; *HRD* haploidentical related donor; *IQR* interquartile range; *BSA* body surface area; *Bu* busulfan; *Flu* fludarabine; *VP* etoposide; *Mel* melphalan; *Cy* cyclophosphamide; *CR* complete remission; *AUC* area under the curve

^a^Two with therapy-related myelodysplastic syndrome, and one with myelodysplastic syndrome, with excess blasts in the HRD group

^b^Two with juvenile myelomonocytic leukemia, one with mixed-phenotype acute leukemia, one with malignant histiocytosis in the UCB group, and two with mixed-phenotype acute leukemia in the HRD group

### Comparisons between the unrelated groups

First, we compared the clinical outcomes of the unrelated 09–13 and 14–18 groups to investigate any differences in HSCT outcomes related to the timing at our institution. The 5-year relapse incidences in the unrelated 09–13 and 14–18 groups were 13.7% and 22.2%, respectively (p = 0.483). Furthermore, the 5-year cumulative incidence rate (CIR) of NRM did not differ significantly between the groups, at 9.8% for unrelated 09–13 versus 5.6% for unrelated 14–18 (p = 0.459). The 5-year event-free survival (EFS) and overall survival (OS) rates were 74.4% and 72.2% (p = 0.956) and 81.9% and 80.5% (p = 0.973), respectively (Supplementary Fig. 1). Given that the same conditioning regimen and similar supportive care were applied in our institution, the outcomes did not differ significantly between unrelated HSCTs performed from 2009–2013 and those performed from 2014–2018.

### Comparisons between the HRD and UCB groups: engraftment, complications, and GVHD

**Fig. 1:**
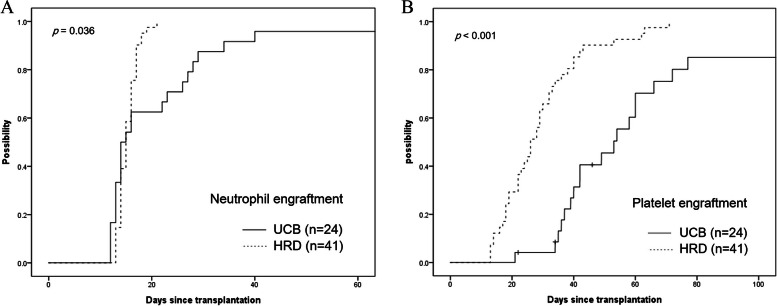
**A** Significantly faster neutrophil engraftment in the HRD group compared with the UCB group (median times to neutrophil engraftment: 15 days [range, 13–21] vs. 14 days [range, 12–40], *p* = 0.036). **B** Platelet engraftment was also faster in the HRD group (median 26 days vs. 46 days, *p* < 0.001)

The median infused total nucleated and CD34 + cell counts were 14.8 × 10⁸/kg and 8.3 × 10⁶/kg in the HRD group, compared with 7.18 × 10⁷/kg/unit and 3.7 × 10^5^/kg/unit (at cryopreservation) in the UCB group. All but three patients in the UCB group received double-unit UCB. The median total nucleated cell count and CD34 + cell count infused from double-unit UCB were 14.28 × 10⁷/kg (range, 6.92–40.5 × 10⁷/kg) and 6.95 × 10^5^/kg (range, 3.25–21.97 × 10^5^/kg), respectively. The median time to neutrophil engraftment was 15 days (range, 13–21 days) in the HRD group and 14 days (range, 12–40 days) in the UCB group. Despite similar medians, neutrophil engraftment was significantly lower in the HRD group than in the UCB group (p = 0.036) (Fig. 1A). Additionally, neutrophil engraftment pattern in the HRD group was more predictive and consistent than that in the UCB group, with all patients in the HRD group achieving neutrophil engraftment, compared with 95.8% in the UCB group (one primary engraftment failure). Platelet engraftment occurred significantly faster in the HRD group than in the UCB group (median 26 days [range, 13–71 days] vs. 46 days [range, 21–77 days], p < 0.001) (Fig. 1B).

The incidence of hepatic veno-occlusive disease, the cytomegalovirus (CMV) antigenemia positivity rate, and antigenemia levels ≥ 10/200,000 cells did not differ significantly between the HRD and UCB groups. However, higher proportions of patients in the UCB group experienced ≥ grade 3 liver enzyme elevation (50.0% vs. 19.5%, p = 0.01) and hyperbilirubinemia (37.5% vs. 2.4%, *p* < 0.001). In contrast, hemorrhagic cystitis > grade 3 occurred more frequently in the HRD group (31.7% vs. 8.3%, *p* = 0.031).

**Fig. 2:**
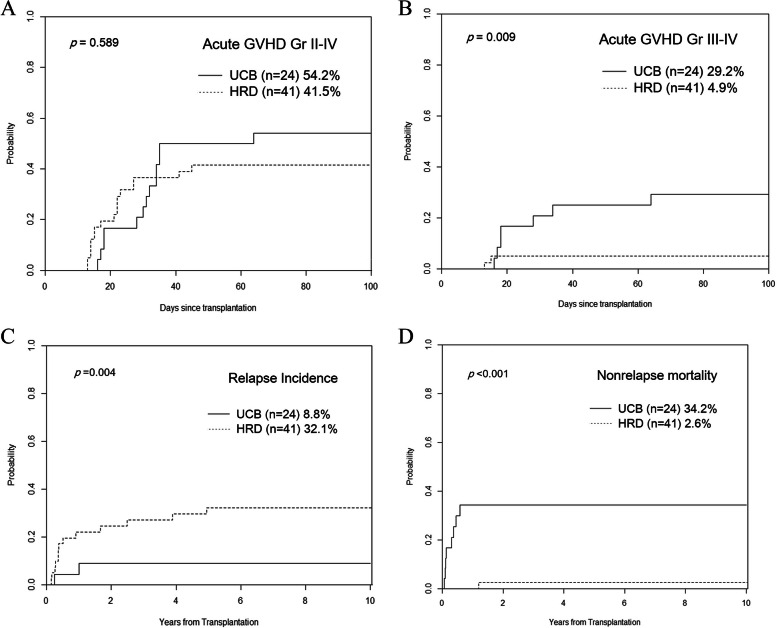
** A** The cumulative incidence rates of acute GVHD grades II–IV were 41.5% in the HRD group versus 54.2% in the UCB group (*p* = 0.589), and **B** grades III–IV were 4.9% in the HRD group versus 29.2% in the UCB group (p = 0.009). **C** Additionally, the relapse incidence was significantly higher in the HRD group compared with the UCB group (*p* = 0.004). **D** However, the cumulative incidence of non-relapse mortality (NRM) was 34.2% in the UCB group, while it was only 2.6% in the HRD group (*p* < 0.001)

Regarding acute GVHD grades II–IV, the CIR did not differ significantly between the HRD and UCB groups (41.5% vs. 54.2%; *p* = 0.589). However, the CIR of acute GVHD grades III–IV in the HRD group was significantly lower than that in the UCB group (4.9% vs. 29.2%, *p* = 0.009) (Figs. 2A and B). The CIRs of moderate-to-severe chronic GVHD were similar between the HRD and UCB groups (14.6% and 12.5%, respectively; p = 0.833).

### Comparisons between the HRD and UCB groups: Relapse, NRM, and survival

The 5-year relapse rate was significantly higher in the HRD group than in the UCB group (32.1% vs. 8.8%; p = 0.004) (Fig. 2C). Conversely, the CIR of the 5-year NRM was markedly lower in the HRD group than in the UCB group (2.6% vs. 34.2%; p < 0.001) (Fig. 2D). Causes of NRM in the UCB group included five cases of pneumonia (with or without acute respiratory distress syndrome), two cases of hepatic veno-occlusive disease accompanied by pulmonary hemorrhage, and one case of hepatic failure. In contrast, the sole cause of NRM in the HRD group was pneumonia associated with acute respiratory distress syndrome.

**Fig. 3:**
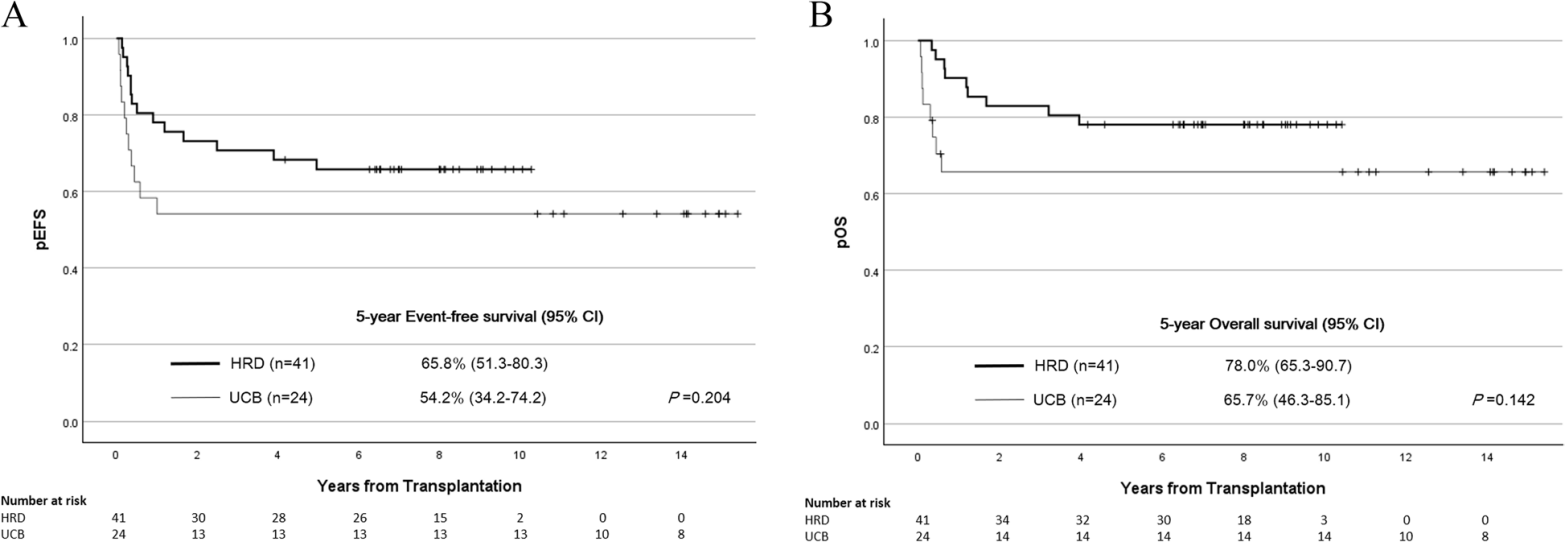
The 5-year EFS rates for the HRD and UCB groups were 65.8% and 54.2%, (p = 0.204) (**A**), while the OS rates were 78.0% and 65.7% (*p* = 0.142) (**B**), respectively

The 5-year EFS and OS rates for the HRD and UCB groups were 65.8% (95% confidence interval [CI] 51.3–80.3%) versus 54.2% (95% CI 34.2–74.2%) (p = 0.204) and 78.0% (95% CI 65.3–90.7%) versus 65.7% (95% CI 46.3–85.1%) (p = 0.142), respectively (Fig. 3C–D). In multivariate analysis, UCB HSCT demonstrated a trend toward worse 5-year EFS compared with HRD HSCT, with a hazard ratio (HR) of 2.63 (95% CI 1.00–6.93; *p* = 0.05). Regarding the 5-year OS rates, disease status at HSCT, specifically second complete remission or higher, was a significant risk factor (HR 3.24, 95% CI 1.25–8.43; p = 0.016) (Table 2).

**Table 2:** Multivariate analysis of EFS and OS rates in the HRD and UCB groups (*N* = 65)

	5-year EFS				5-year OS			
	Univariate		Multivariate		Univariate		Multivariate	
	Probability ± SE	*p*-value	HR (95% CI)	*p*-value	Probability ± SE	*p*-value	HR (95% CI)	*p*-value
HSCT type		0.204		0.05		0.142		0.113
HRD (n = 41)	65.8 ± 7.4%		1		78.0 ± 6.5%		1	
CBT (n = 24)	54.2 ± 10.2%		2.63 (1.00–6.93)		65.7 ± 9.9%		2.17 (0.83–5.65)	
Sex		0.468				0.75		
Male (n = 32)	65.6 ± 8.4%				74.4 ± 7.8%			
Female (n = 33)	57.6 ± 8.6%				72.4 ± 7.8%			
Age at HSCT (years)		0.232		0.224		0.742		
2–10 (n = 27)	51.9 ± 9.6%		1		69.1 ± 9.1%			
< 2 (n = 15)	80.0 ± 10.3%		0.30 (0.08–1.18)	0.084	80.0 ± 10.3%			
> 10 (n = 23)	60.6 ± 10.3%		0.82 (0.30–2.27)	0.702	73.9 ± 9.2%			
Disease status		0.009		0.104		0.053		0.016
CR1 (n = 46)	71.7 ± 6.6%		1		84.4 ± 5.4%		1	
≥ CR2 (n = 19)	36.8 ± 11.1%		2.24 (0.85–5.94)		63.2 ± 11.1%		3.24 (1.25–8.43)	
Diagnosis		0.241		0.187		0.723		
ALL (n = 26)	65.4 ± 9.3%		1		76.9 ± 8.3%			
AML (n = 24)	54.2 ± 10.2%		2.07 (0.77–5.60)	0.151	69.5 ± 9.7%			
Malignant lymphoma (n = 6)	83.3 ± 15.2%		0.49 (0.06–4.04)	0.507	83.3 ± 15.2			
Myelodysplastic syndrome (n = 4)	25.0 ± 21.7%		0.73 (0.08–6.30)	0.771	50.0 ± 25.0%			
Others (n = 5)	80.0 ± 17.9%		3.81 (0.85–17.53)	0.086	80.0 ± 17.9%			

*ALL* acute lymphoblastic leukemia; *AML* acute myeloid leukemia; *AUC* area under the curve; *CI* confidence interval; *CR* complete remission; *EFS* event-free survival; *HR* hazard ratio; *HRD* haploidentical related donor; *HSCT* hematopoietic stem cell transplantation; *OS* overall survival; *SE* standard error; *UCB* umbilical cord blood

## Discussion

The results of this retrospective study demonstrated that unmanipulated HRD HSCT using PTCy was associated with faster engraftment and a lower incidence of NRM in pediatric patients with hematologic malignancies. These benefits contributed to improved EFS and OS rates compared with UCB HSCT using ATG in the context of an intensively pharmacokinetically monitored busulfan-based myeloablative conditioning regimen. To our knowledge, this is the first study in Korea to compare HRD HSCT with PTCy and UCB HSCT in pediatric patients, despite previous studies reporting favorable outcomes for T-cell-depleted or ATG-based HRD HSCT compared with UCB HSCT in pediatric populations [19–21].

Most studies comparing HRD and UCB HSCT, have focused on adults. While most reported better outcomes for HRD HSCT, including lower rates of GVHD and NRM[13, 15, 17, 18], limitations due to the retrospective study design and patient heterogeneity have made definitive conclusions challenging. A prospective randomized trial using a myeloablative regimen in adults with hematologic malignancies showed improved outcomes for HRD HSCT with PTCy compared with ATG-containing single-unit UCB HSCT [16]. Conversely, a recent randomized trial in the US (BMT CTN 1101) demonstrated similar 2-year progression-free survival rates between adults administered reduced-intensity, unmanipulated HRD HSCT or PTCy with double-unit UCB HSCT [27]. Given the challenges of patient accrual and variability in institutional HSCT practices, conducting a prospective randomized comparative study in pediatric patients may be difficult. Owing to the limited data in pediatric populations, this study holds potential clinical significance by offering insights into the selection of alternative donors for unmanipulated HRD HSCT with PTCy.

The results of this study also demonstrated that a lower cumulative incidence of NRM and grade III–IV acute GVHD in the HRD group contributed to favorable EFS and OS rates compared with those in the UCB group. In the UCB group, the primary causes of NRM were infectious complications or hepatic toxicities, mostly occurring within seven months after HSCT. Notably, only one case of NRM occurred in the HRD group. Predictable neutrophil engraftment in the HRD group likely contributed to the reduction in early infectious complications after transplantation compared with the UCB group. Although the busulfan AUC in the UCB group was lower than that in the HRD group, this finding suggests that the higher NRM rates in the UCB group were not attributable to busulfan exposure. Additionally, while the reduced NRM rate in the HRD group could potentially be attributed to improved supportive care over time, the NRM rates did not differ significantly between the unrelated 09–13 and 14–18 cohorts. The introduction of PK-guided busulfan in our myeloablative conditioning regimen may have also contributed to the reduced HSCT-related toxicity. Although the relapse incidence was lower in the UCB group than in the HRD group, caution is warranted when interpreting this finding as high early NRM in the UCB group serves as a competing risk factor for relapse.

Given the recent advancements in HRD HSCT with PTCy, its relatively straightforward learning curve, and faster donor acquisition and engraftment times that facilitate more predictable clinical courses[11], our institution has prioritized HRD HSCT over UCB in cases without matched donors since 2016. This shift makes direct comparisons between HRD and UCB HSCT within the same timeframe challenging. During the transitional period from 2014 to 2016, UCB was the preferred alternative donor source when matched donors were not available. However, from 2016 onward, we adjusted our donor selection criteria to focus on reducing early NRM rates. Although outcomes did not differ significantly across the unrelated donor groups based on the period of treatment at our institution, caution should be exercised when comparing HRD and UCB HSCT outcomes in the pediatric population.

This study has several limitations. First, the retrospective comparison between the two groups was influenced by differing timelines. Notably, the UCB group received busulfan-based chemotherapy with ATG. Previous research has shown that total body irradiation offers better outcomes than chemoconditioning in pediatric patients with acute lymphoblastic leukemia undergoing UCB-HSCT[28], highlighting opportunities for further optimization. Additionally, while haploidentical donors with the KIR B haplotype were prioritized, a younger donor age was identified as a critical prognostic factor for HRD HSCT with PTCY [29, 30]. This factor is particularly relevant for pediatric patients who may have the option of receiving stem cells from a parent or sibling.

In summary, the results of this study suggest that HRD-HSCT with PTCy, combined with an intensive PK-monitored, targeted busulfan-based myeloablative conditioning regimen, is a safe and promising alternative for pediatric patients with hematological diseases who lack an HLA-matched donor. Nevertheless, prospective studies in children and adolescents are essential to enable more robust comparisons between these two stem cell sources.

## Supplementary Information

Supplementary Material 1.

## Data Availability

The datasets generated and analyzed during the current study are available from the corresponding author on reasonable request.

## Abbreviations

HRD: Haploidentical related donor
UCB: Umbilical cord blood
HSCT: Hematopoietic stem cell transplantation
GVHD: Graft-versus-host disease
NRM: Non-relapse mortality
PTCy: Post-transplantation cyclophosphamide
ATG: Anti-thymocyte globulin
PK: Pharmacokinetics
AUC: Area under the curve
CIR: Cumulative incidence rate
EFS: Event-free survival
OS: Overall survival
HR: Hazard ratio
CI: Confidence interval
