# Supplementary material for: Comparing haploidentical transplantation with post-transplantation cyclophosphamide and umbilical cord blood transplantation using targeted busulfan in children and adolescents with hematologic malignancies
Source: Blood Res. 2025 Jan 23;60(1):7. doi: 10.1007/s44313-025-00057-7 (PMC11757842; doi:10.1007/s44313-025-00057-7)

**Supplementary Table 1. Patient characteristics including the Unrelated groups.**

|  |  | Unrelated 09-13 (n=51) | Unrelated 14-18 (n=36) | UCB  (n=24) | | HRD (n=41) | *p* value |
| --- | --- | --- | --- | --- | --- | --- | --- |
| Median age, Years (IQR) | | 11.3 (5.4–14.8) | 8.6 (3.0–12.7) | 2.4 (1.4–6.1) | 11.3 (5.8–14.2) | | <0.001 |
| Sex, No. (%) | |  |  |  |  | | 0.121 |
|  | Male | 26 (51.0%) | 22 (61.1%) | 8 (33.3%) | 24 (58.5%) | |  |
|  | Female | 25 (49.0%) | 14 (38.9%) | 16 (66.7%) | 17 (41.5%) | |  |
| Median BSA, m^2^ (IQR) | | 1.20 (0.74–1.51) | 1.0 (0.64–1.37) | 0.55 (0.48–0.80) | 1.11 (0.67–1.54) | | <0.001 |
| Median Body weight, kg (IQR) | | 38.0 (18.2–51.3) | 28.8 (15.5–45.3) | 12.23 (9.8–21.1) | 31.2 (15.7–50.6) | | <0.001 |
| Diagnosis, No. (%) | |  |  |  |  | | 0.172 |
|  | Acute lymphoblastic leukemia | 22 (43.1%) | 19 (52.8%) | 10 (41.7%) | 16 (39.0%) | |  |
|  | Acute myeloid leukemia | 23 (45.1%) | 8 (22.2%) | 10 (41.7%) | 14 (34.1%) | |  |
|  | Myelodysplastic syndrome† | 1 (2.0%) | 0 (0.0%) | 0 (0.0%) | 3 (7.3%) | |  |
|  | Malignant lymphoma | 2 (3.9%) | 6 (16.7%) | 0 (0.0%) | 6 (14.6%) | |  |
|  | Others* | 3 (5.9%) | 3 (8.3%) | 4 (16.7%) | 2 (4.9%) | |  |
| Conditioning regimen | |  |  |  |  | | <0.001 |
|  | Bu+Flu | 15 (29.4%) | 4 (11.1%) | 8 (33.3%) | 0 (0.0%) | |  |
|  | Bu+Flu+VP | 34 (66.7%) | 32 (88.9%) | 14 (58.3%) | 0 (0.0%) | |  |
|  | Bu+Flu+Mel | 2 (3.9%) | 0 (0.0%) | 2 (8.3%) | 0 (0.0%) | |  |
|  | Bu+Flu+Cy | 0 (0.0%) | 0 (0.0%) | 0 (0.0%) | 41 (100.0%) | |  |
| Status | |  |  |  |  | | 0.715 |
|  | CR1 | 36 (70.6%) | 29 (80.6%) | 17 (70.8%) | 29 (70.7%) | |  |
|  | ≥CR2 or persistence | 15 (29.4%) | 7 (19.4%) | 7 (29.2%) | 12 (29.3%) | |  |
| Infused busulfan AUC, mg x h/L (IQR) | | 75.4 (74.2–76.9) | 74.1 (73.3–75.5) | 73.7 (72.0–75.2) | 74.5 (74.0–76.0) | | 0.01 |
| Median follow-up years (IQR) | | 11.1 (5.5-13.0) | 7.3 (5.7-9.1) | 10.9 (0.4-14.1) | 7.0 (4.4-8.7) | | 0.006 |

UCB, umbilical cord blood; HRD, haploidentical related donor; IQR, interquartile range; No., Number; BSA, body surface area; Bu, busulfan; Flu, fludarabine; VP, etoposide; Mel, melphalan; Cy, cyclophosphamide; CR, complete remission; and AUC, area under the curve

† 1 Myelodysplastic syndrome with excess blasts in Unrelated A group, 2 therapy-related myelodysplastic syndromes, and 1 myelodysplastic syndrome, with excess blasts in the HRD group.

* 2 Mixed-phenotype acute leukemia and 1 NK cell leukemia each in the Unrelated A and B groups, 2 Juvenile myelomonocytic leukemia, 1 Mixed-phenotype acute leukemia, and 1 Malignant histiocytosis in the UCB group, and 2 Mixed phenotype acute leukemia in the HRD group.

**Supplemantary Figure**

The 5-year EFS (A) and OS (B) rates for all groups, including the unrelated donor groups, are presented.

**1A**


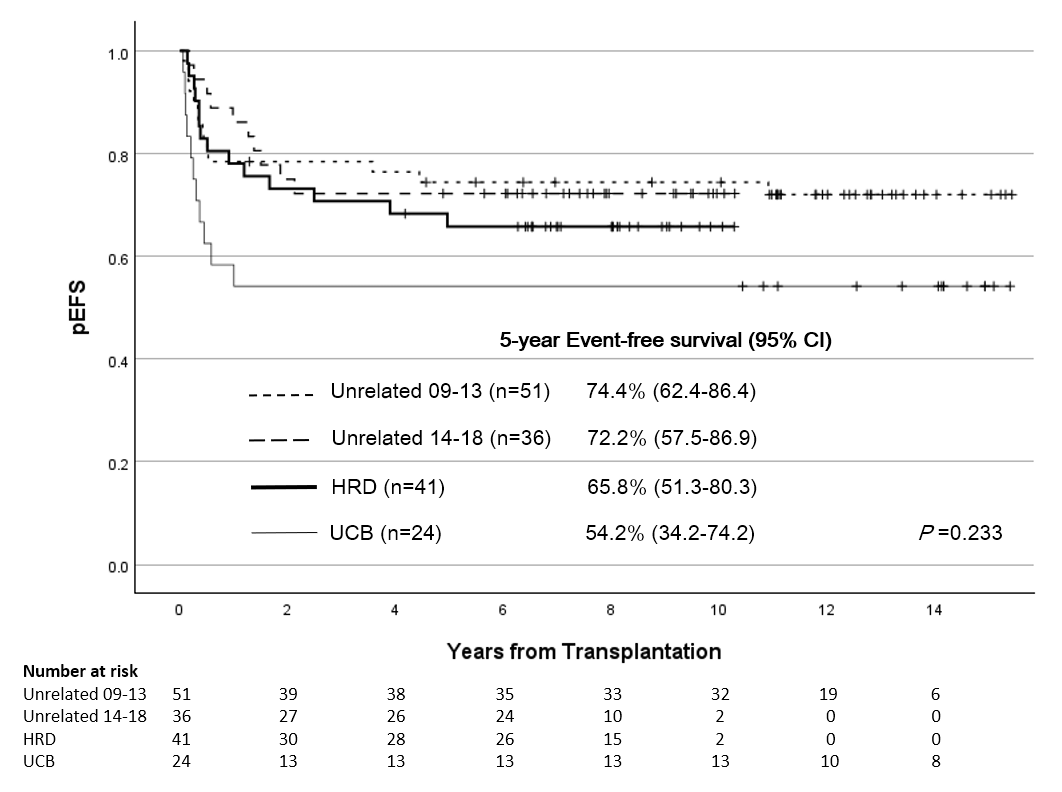


**Supplemantary Figure 1B**


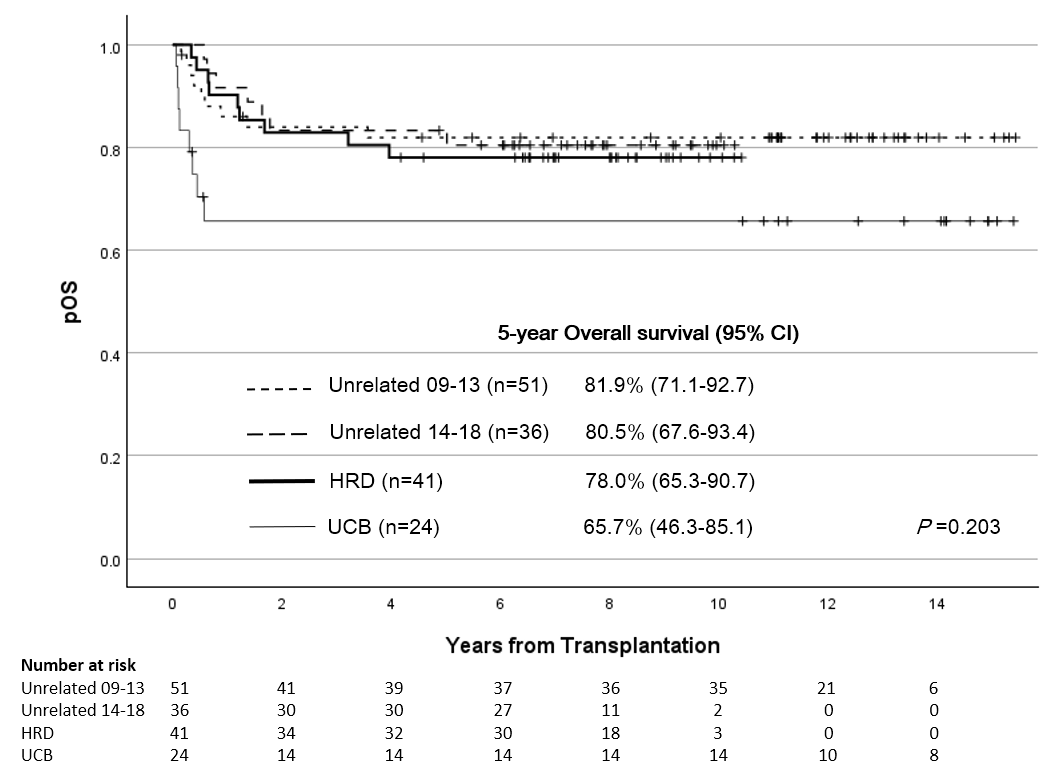

Supplement: Supplementary file 1 — Supplementary Material 1. [file 44313_2025_57_MOESM1_ESM.docx]
